# The cerebellum during provocation and aggressive behaviour: A 7 T fMRI study

**DOI:** 10.1162/imag_a_00044

**Published:** 2023-12-14

**Authors:** Elze M.L. Wolfs, Wietske Van der Zwaag, Nikos Priovoulos, Jana Klaus, Dennis J.L.G. Schutter

**Affiliations:** Department of Experimental Psychology, Helmholtz Institute, Utrecht University, Utrecht, The Netherlands; Spinoza Centre for Neuroimaging, The Royal Netherlands Academy of Arts and Sciences, Amsterdam, The Netherlands; Computational Cognitive Neuroscience and Neuroimaging, Netherlands Institute for Neuroscience, Amsterdam, The Netherlands; Department of Biomedical Engineering & Physics, Amsterdam UMC, University of Amsterdam, Amsterdam, The Netherlands

**Keywords:** anger, cerebellum, corticosteroid hormones, functional magnetic resonance imaging, provocation, ultra-high field fMRI

## Abstract

Increasing empirical evidence points towards the involvement of the cerebellum in anger and aggressive behaviour. However, human functional neuroimaging studies so far have emphasised the involvement of subcortical and cortical regions, rather than examining the contributions of the cerebellum. In the present study, 7 T functional magnetic resonance imaging (fMRI) was used to assess cerebellar activation during provocation and aggressive behaviour elicited by the Point Subtraction Aggression Paradigm in 29 healthy adult volunteers. Provocations resulted in left posterior cerebellar activation, while right posterior cerebellar activation was associated with aggressive behaviour. Our findings confirm the involvement of distinct and lateralised non-motor related cerebellar areas during provocation and aggressive behaviour. This study adds to the growing recognition of the posterior cerebellar regions in emotion- and cognition-dedicated processes and to the role of the little brain in human aggression.

## 1 Introduction

Aggression can be defined as behaviour with the intent to cause harm or injury to a person or object in response to provocation or frustration (Anderson & Bushman, 2002; Berkowitz, 1965). Aggressive behaviour may prove useful in removing threats from the environment, but aggression typically has a negative connotation (e.g., exaggerated and pathological) (Siever, 2008). The neurobiological circuit of aggression is composed of several cortical and subcortical regions, including the hypothalamus, amygdala, periaqueductal grey (PAG), and medial prefrontal cortex (mPFC), which are associated with the motivational, emotional, and cognitive aspects of aggressive behaviour (Davidson et al., 2000; LeDoux, 2012; Lischinsky & Lin, 2020; Panksepp & Biven, 2012).

In addition, several lines of evidence point towards involvement of the cerebellum in aggressive behaviour (for a theoretical framework, see Kruithof et al., 2022): In animals, electrical stimulation of the deep cerebellar nuclei (DCN), the main output structure of the cerebellar cortex (Habas et al., 2016), has been shown to result in sham rage and attacks (Reis et al., 1973; Zanchetti & Zoccolini, 1954). In addition, increased aggressive behaviour has been observed after optogenetic deactivation of inhibitory Purkinje cells in the cerebellar midline (vermis) in mice, arguably due to indirect activation of the DCN (Jackman et al., 2020). Moreover, patients with cerebellar lesions or malformations have shown emotion dysregulation, impulsivity, blunted affect, and aggression (e.g., Cosme-Cruz et al., 2022; Hoche et al., 2018; Levisohn et al., 2000; Schmahmann & Sherman, 1998; Schutter et al., 2021; Tessier et al., 2015; Tonna et al., 2014). Furthermore, grey matter volumetry and voxel-based morphometry studies using structural neuroimaging have found evidence that psychiatric and neurological patients who display high levels of aggression, impulsivity, and anger show structural abnormalities in the vermis (Bertsch et al., 2013; De Brito et al., 2009; Huebner et al., 2008; Kuhlmann et al., 2013; Lee et al., 2011; Leutgeb et al., 2015) and bilateral posterior lobules (e.g., Bertsch et al., 2013; Coccaro et al., 2016; Okada et al., 2015; Puri et al., 2008; Soloff et al., 2008; J. Zhang et al., 2018, 2019). These findings are complemented by research in healthy volunteers, where reduced grey matter volumes in the right posterior lobe are associated with higher aggression scores and increased vermal grey matter volumes are linked to higher impulsivity scores (Wolfs, Klaus, et al., 2023). Finally, the cerebellum shows structural (e.g., Cacciola et al., 2019; Kamali et al., 2018) and functional (e.g., Leutgeb et al., 2016; Roy et al., 2014; Wolfs, van Lutterveld, et al., 2023) connections to the aforementioned subcortico-cortical aggression circuit, arguably facilitating cerebellar input to the PAG, amygdala, hypothalamus, and prefrontal regions. Together, these findings provide a functional neuro-anatomical basis for a cerebellar role in aggressive behaviour that focuses on the vermis and posterior hemispheres.

Through its reciprocal connections to the hypothalamus (Çavdar et al., 2018; Dietrichs, 1984; Haines et al., 1984; Kamali et al., 2018), the cerebellum can exert an influence on the hormonal axes and aggressive behaviour forming a cerebello-hypothalamic-pituitary-adrenal (cerebello-HPA) axis (Schutter, 2012, 2020). Previous endocrinological studies have shown that the steroid hormones testosterone and cortisol are associated with aggressive behaviour (Montoya et al., 2012; Terburg et al., 2009; Van Honk et al., 2010). Testosterone and cortisol are the main end-products of the hypothalamic-pituitary-gonadal (HPG) and HPA axis, respectively (Johnson et al., 1992). Acute increases of endogenous testosterone levels can sensitise the hypothalamus and midbrain structures and facilitate fight-related motivational tendencies associated with anger and aggression, while cortisol can downregulate the activating effects of testosterone and desensitises the subcortical components of the aggression circuit (Hermans et al., 2008). Through the mutually inhibitory effects of the HPG and HPA axes, the system may bias itself towards an imbalance between hormone levels, that is, high testosterone and low cortisol or vice versa (Montoya et al., 2012). An imbalance towards testosterone, that is, a higher ratio between testosterone (T) and cortisol (C), has been associated with higher aggressive behaviour and this T/C ratio is suggested to provide a better marker for aggression than the steroid hormones separately (Manigault et al., 2019; Platje et al., 2015; Popma et al., 2007; Terburg et al., 2009). In addition to its connections with the hypothalamus, the cerebellum can be considered a target region for steroid hormonal modulation through the presence of corticoid and androgen receptors in the cerebellar cortex (Sánchez et al., 2000; Webster et al., 2002).

Functional magnetic resonance imaging (fMRI) studies in humans that employ laboratory aggression paradigms have proven valuable to elucidate the neural correlates of human aggression (Fanning et al., 2017; Nikolic et al., 2022; Wong et al., 2019). Previous fMRI work has demonstrated activation of key brain regions implicated in aggression during provocation from a fictional opponent, including the amygdala and prefrontal cortex (e.g., Chen et al., 2021; da Cunha-Bang et al., 2017; Kaltsouni et al., 2021; Skibsted et al., 2017). Despite the available empirical evidence, the cerebellum is not typically considered a region of interest in fMRI studies on aggressive behaviour. The limited amount of laboratory aggression paradigms that have reported cerebellar activation in their whole brain activation were recently summarised in a meta-analysis (Klaus & Schutter, 2021). Results (*k* = 10) showed evidence for cerebellar activation of the right posterior lobe and bilateral anterior lobes. In addition, recent evidence was found for the involvement of bilateral Crus I-II while viewing threatening faces during an aggression task (Bertsch et al., 2022). Altogether, fMRI studies that show cerebellar activation during provocation or aggressive behaviour remain scarce. This may, in part, be due to not having included the cerebellum as a region of interest, exclusion of the cerebellum in the field of view or to low signal-to-noise ratio (SNR) in the posterior fossa (Diedrichsen et al., 2010; Fair, 2018). With 7 T fMRI, increased SNR and blood-oxygen-level-dependent (BOLD) sensitivity may reveal more subtle BOLD effects (Cai et al., 2021; van der Zwaag et al., 2009).

The present study therefore investigated cerebellar activation associated with provocation and aggressive behaviour using 7 T fMRI in healthy volunteers. In keeping with previous neuroimaging and stimulation studies, we hypothesised activation of the posterior lobules and vermis during provocation (i.e., points were stolen by a fictitious opponent) or aggressive behaviour (i.e., participants stole points from a fictitious opponent). Additionally, relations between cerebellar activation, aggressive behaviour, steroid hormones, state anger, trait aggression, and trait impulsivity were explored. We anticipated that cerebellar activation during provocation and aggressive behaviour would be positively correlated with trait aggression, impulsivity, and T/C ratios. Additionally, increased aggressive behaviour was expected to correlate with higher trait aggression. Finally, higher T/C ratios were expected to correlate with increased aggressive behaviour and with higher levels of state anger, trait aggression, and impulsivity.

## 2 Methods

### 2.1 Participants

Thirty healthy right-handed volunteers between 18-35 years old participated in the study that took place at the Spinoza Centre for Neuroimaging in Amsterdam, the Netherlands. Participants were excluded if they had current or previous neurological or psychiatric complaints, were not MRI compatible (e.g., claustrophobia, electronic implants, pregnancy, metal in their body), or used psychotropic medication or recreational drugs. Written informed consent was obtained. Participants received travel reimbursement and monetary compensation for participation. The study was approved by the medical ethical committee from the University Medical Center Utrecht (NL77559.041.21) and performed in accordance with the Declaration of Helsinki. Out of the 30 participants, one person was excluded for excessive motion (mean absolute displacement > 1 mm) during scanning, leading to a final sample of 29 participants.

### 2.2 Aggression task

The Point Subtraction Aggression Paradigm (PSAP) is a validated laboratory task, in which participants play a game against a fictional opponent. In the present study, participants were told that the opponent was playing online. The goal for the participants is to earn as many points as possible (Cherek et al., 1997). Participants can also choose to steal points from their opponent. As participants do not receive the stolen points, this act of retaliation can be considered a form of aggressive behaviour intended to harm an opponent who provokes them. The task was adapted from previous versions (Geniole et al., 2017; Kose et al., 2015) implemented in E-Prime v3.0 (https://www.pstnet.com/eprime.cfm).

**Fig. 1.**
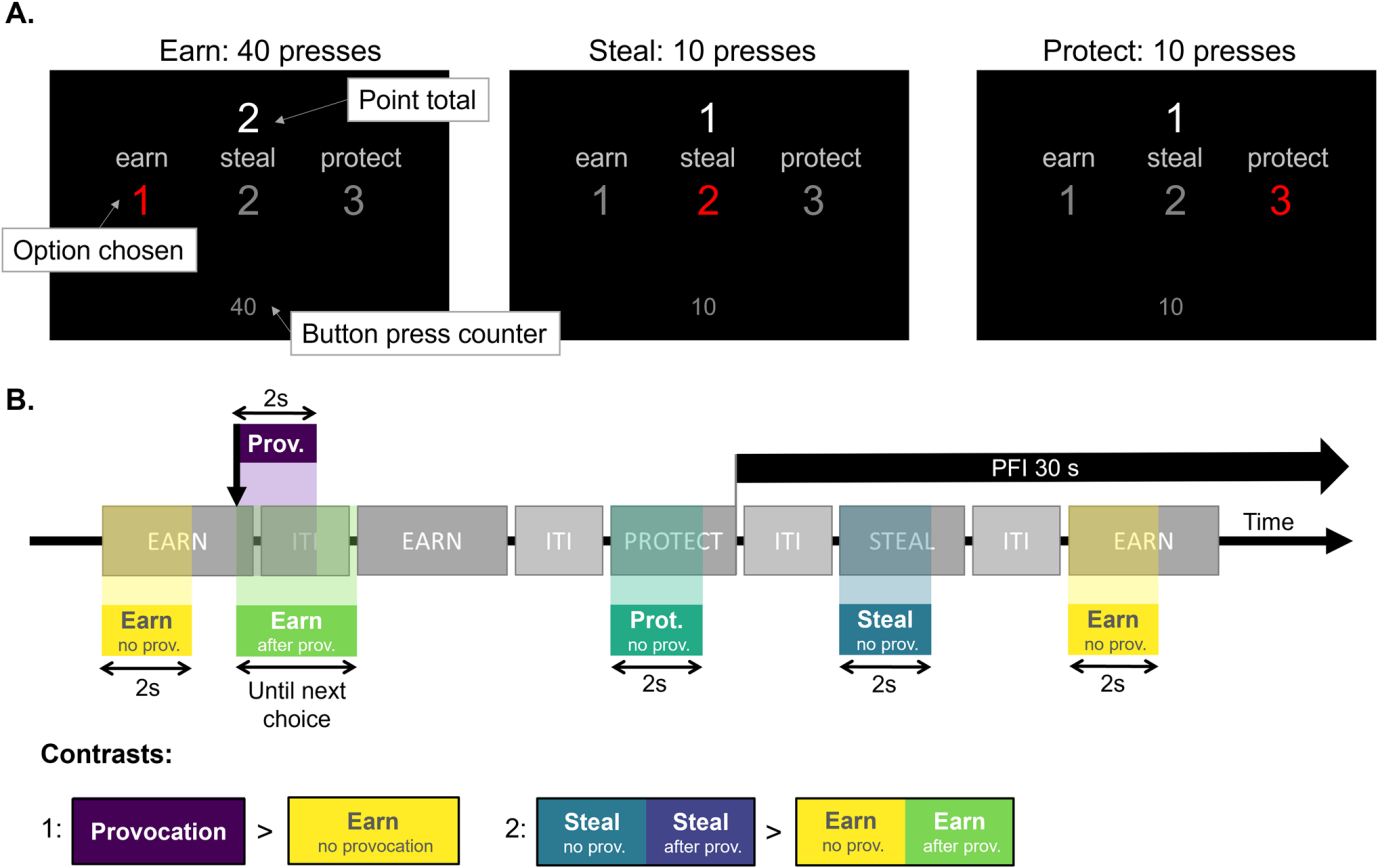
Point Subtraction Aggression Paradigm set-up. (A) Presentation of the three options for each trial: earn, steal, and protect. (B) Possible schematic timeline of the task, including visualisation of the onsets and durations of the regressors used in the Provocation > Earn and Steal > Earn contrasts. The ITI was of variable 4-6 second duration. Abbreviations: ITI = Inter-Trial Interval; no prov. = no preceding provocation; PFI = Provocation-Free Interval; prot. = protect; prov. = provocation; s = seconds.

During the task, three options were continuously presented to the participant: participants could gain points (earn option), steal points from the opponent (steal option), or prevent steals by the opponent (protect option) (Fig. 1A). To earn a point, 40 button presses had to be completed, while 10 button presses had to be completed to steal a point or protect the point total. When a choice for one of the options was made, the participants had to complete the total number of button presses (40 or 10) before switching to another option. Between choices, participants waited for 4-6 seconds (inter-trial interval, ITI) (Fig. 1B). Throughout the task, participants could keep track of their point total, current choice made, and number of times a given button had already been pressed (out of 10 or 40, depending on their choice) on the computer screen. When a point was earned, the point total increased by one and positive visual feedback was provided (“+” symbols flashed around the point total for 1000 ms). Throughout the task, participants could have points stolen by the fictional opponent (i.e., a provocation). When a point got stolen, one point was subtracted and negative visual feedback was provided (flashing “-“ symbols shown for 1000 ms). Provocations occurred randomly every 6-45 seconds, on average 10 times per 9-minute run. At the start of the task, their point total was protected for 45 seconds (provocation-free interval, PFI). When participants completed the “steal” or “protect” option, a PFI of 30 seconds occurred. Participants, however, were only aware of a protective effect for the “protect” option and did not know the length of the PFI beforehand, but thought this was of variable duration.

Before scanning, participants practised the task outside of the scanner for 2 minutes. Here, they were given the opportunity to ask questions and resolve any uncertainties. In the scanner, a button box was placed under the thumb, index, and middle finger of the right hand, corresponding to the three options during the task. Their monetary reward was 5 eurocents per point earned, which was rounded up to a maximum of 3 euros for each participant. In the scanner, participants performed two runs that each lasted 9 minutes. In our sample of 29 participants, three participants performed just one run due to technical problems. After the PSAP, participants reported what tactic they employed and what they thought of their opponent and their opponent’s tactic. Afterwards, they were informed of the aim of the task and that their opponent was a computer.

### 2.3 Behavioural assessment of anger, aggression, and impulsivity

#### 2.3.1 State Anger

The State Anger (SA) scale of the State-Trait Anger Scale (STAS; Spielberger et al., 1983; Dutch version: Van der Ploeg et al., 1982) was administered to measure participants’ self-reported emotional state and feelings such as anger, irritation, and rage. The SA consists of 10 items with possible answers ranging from “not at all” to “extremely” on a four-point scale. The SA was administered before and after the scanning session to acquire a baseline emotional state before the task as well as to quantify the change in SA after being in the scanner and performing two rounds of the PSAP.

#### 2.3.2 Trait aggression

The Buss-Perry Aggression questionnaire (BPA; Buss & Perry, 1992; Dutch version: Meesters et al., 1996) was administered after the scanning session to obtain a self-reported measure of trait anger and aggression. The BPA consists of four subscales: physical aggression (9 items), verbal aggression (5 items), anger (7 items), and hostility (8 items). Every question is answered on a five-point scale ranging from “extremely uncharacteristic of me” to “extremely characteristic of me.”

#### 2.3.3 Trait impulsivity

The Barratt Impulsiveness Scale (BIS-11; Patton et al., 1995; Dutch version: Lijffijt & Barratt, 2005) was administered after the scanning session as a self-reported measure of impulsive behaviour. The BIS-11 consists of three subscales: attentional impulsivity (8 items), motor impulsivity (11 items), and non-planning impulsivity (11 items). Every question is answered on a four-point scale ranging from “seldom/never” to “almost always.”

### 2.4 Steroid hormones

Saliva samples were collected directly before the MRI scanning session by having participants spit in a saliva vial. Participants were instructed to not eat or drink anything besides water for 2 hours before the study. Immediately after collection, the samples were stored in a freezer at -80°C. Testosterone and cortisol levels were determined from the saliva samples by the Central Diagnostic Laboratory (CDL) at the University Medical Center Utrecht.

Salivary testosterone levels were assessed in duplicate using an in-house competitive radio-immunoassay employing a polyclonal anti-testosterone-antibody (Dr. Pratt AZG 3290). [1,2,6,7-^3^H]-Testosterone (NET370250UC, PerkinElmer) was used as a tracer following chromatographic verification of its purity. The lower limit of detection was 10 pmol/L. Inter-assay variation was 9.1, 4.3, and 5.6% at 95, 200, and 440 pmol/L, respectively. Intra-assay variation was 7.2 and 2.5% at 38 and 92 pmol/L, respectively (*n* = 10).

Salivary cortisol levels were determined by liquid chromatography-tandem mass spectroscopy (LC–MS). 100 µL saliva was mixed with internal standards Cortisol-9,11,12,12-D4 and Cortisone-D8 using MRQ30-vials (Supelco). Samples were evaporated under N_2_ at 70 °C. Residues were reconstituted in 30 µL 80% methanol and quantified by LC–MS/MS. Calibrator solutions were prepared from Sigma-Aldrich hydrocortisone and cortisone stock solutions. The UHPLC-MS/MS system consisted of an Ultimate 3000 UPLC system coupled with an APCI TSQ Quantiva mass spectrometer (ThermoElectron Corp, West Palm Beach, FL). An Acquity UPLC C18 150 × 2.1 mm, 1.7 µm (Waters) column was used with a gradient elution of water/methanol containing 0.1% formic acid for separating cortisol and cortisone. The lower limit of detection was 0.5 nmol/L. Day-to-day imprecision was < 6% at 1.5 and 23 nmol/L for cortisol, and <10% at 6 and 30 nmol/L for cortisone. Intra-assay variation was <2% at 1.2 and 9 nmol/L for cortisol, and <3% at 1.1 and 24 nmol/L for cortisone.

### 2.5 fMRI: image acquisition

All images were acquired on a 7 T Philips Achieva scanner (Philips, Best, The Netherlands) equipped with an 8Tx/32Rx rf coil (Nova Medical, Wilmington USA). As an anatomical reference, T1-weighted images were acquired with Magnetization Prepared 2 Rapid Acquisition Gradient Echoes (MP2RAGE (Marques et al., 2010); TE/TR/TR_mp2rage_ = 2.5 ms/6.2 ms/5.5 s, SENSE_y/z_ = 1.8/1.8, flip angle = 8/5º, TI = 800/2700 ms, voxel size = 0.8 × 0.8 × 0.8 mm, FOV = 230 × 230 × 186 mm, scanning time = 638 s). Functional images were acquired while the participants performed the PSAP with 3D echo planar imaging (3D-EPI, TE/TR_vol_ = 17 ms/1.3 s, SENSE_y/z_ = 2.60/3.27, flip angle = 20^o^, voxel size = 1.8 × 1.8 × 1.8 mm, FOV = 200 × 200 × 176 mm, scanning time = 2 × 540 s). To optimise 7 T cerebellar signal, universal Kt-points pulses were used to reduce B1 inhomogeneity (Gras et al., 2017; Oliveira et al., 2021; Roos, 2019). After each 3D-EPI fMRI acquisition, five more volumes were acquired with reversed phase encoding direction to allow distortion correction.

### 2.6 fMRI: image preprocessing

All PAR/REC image files were converted to NIfTI with dcm2niix (Li et al., 2016). Structural T1-weighted data were obtained from the two inversion time images using MATLAB (The Mathworks, Inc.; https://github.com/JosePMarques/MP2RAGE-related-scripts). Preprocessing of NIfTI images was performed in FSL (FMRIB’s Software Library, Oxford, UK) version 6.0.2 (Jenkinson et al., 2012): Following distortion correction using FSL’s topup (Andersson et al., 2003; Smith et al., 2004), data were processed with fMRI Expert Analysis Tool v6.00 (FEAT; Woolrich et al., 2001). Functional scans were motion-corrected with MCFLIRT (Jenkinson et al., 2002), smoothed at 3 mm Full Width at Half Maximum (FWHM) with SUSAN (Smith & Brady, 1997) and a brain mask was created from the mean functional volume with the Brain Extraction Tool (BET; Smith, 2002). Further, functional data were normalised for higher-level analyses by a single scaling factor (“grand mean scaling”). From the structural scans, non-brain structures were removed with BET. Functional scans were realigned to the structural scan and MNI152 standard space by affine registration using FLIRT (Jenkinson & Smith, 2001; Jenkinson et al., 2002). ICA-AROMA (Pruim et al., 2015), high-pass filter, and nuisance regression were used to remove noise from the data. ICA-AROMA was used to generate 100 independent components, which were extracted from the functional data based on spatial patterns, frequency spectra, and time series. The classification (signal vs. noise) of each component was checked manually and was reclassified if necessary (Griffanti et al., 2017). ICA-AROMA’s non-aggressive denoising was performed based on these labels. Furthermore, cerebrospinal fluid (CSF) and white matter (WM) signals were segmented from the structural scans using FAST (Zhang et al., 2001). Signals from the functional scans in the CSF and WM were used as nuisance regressors. Finally, a high-pass filter at 0.01 Hz was used for data preprocessing.

### 2.7 Analyses

#### 2.7.1 fMRI: single-subject analyses

A general linear model (GLM) was used to model BOLD activation per participant per task run (Beckmann et al., 2003; Ogawa et al., 1990). Both 9-minute task runs were modelled separately and combined using fixed effect modelling in FEAT (Woolrich et al., 2001). In the design matrix for each run, onset times of provocations and each option (i.e., earn, steal, and protect) in the PSAP were included. Per option, two regressors were added: One for the trials directly following a provocation and one for the trials that did not follow a provocation (Fig. 1B). For the trials following a provocation, onset times of the provocations were used instead of the start of earning/stealing/protecting, to model the reaction and decision making after the provocation. These events lasted until the next option was chosen. Earn, steal, and protect trials without preceding provocation were modelled with 2-second durations after the start of the option (onset time). This duration was taken to be a representative duration of a block of button presses for the shorter options (i.e., steal and protect). Duration for provocations was also modelled as 2 seconds. Furthermore, the onset times for button presses were included as a separate regressor. Due to the quick nature of the button presses, duration was set to zero seconds. All regressors were convolved with a hemodynamic response function and its temporal derivative (Friston et al., 1998). Temporal autocorrelation was removed by FILM pre-whitening and the GLM was fitted voxel-wise (Woolrich et al., 2001).

Similar to previous studies that used the PSAP in an fMRI set-up (da Cunha-Bang et al., 2017; Kaltsouni et al., 2021; Skibsted et al., 2017), contrast images of parameter estimates were generated for BOLD activation after a provocation compared to earning a point (i.e., choosing option “1”) and stealing a point (i.e., choosing option “2”) compared to earning a point. These two contrasts are hereafter referenced to as Provocation > Earn and Steal > Earn, respectively (Fig. 1B). For the Steal > Earn contrast, earn trials with and without a preceding provocation were used in both conditions. However, for the Provocation > Earn contrast, earn trials following a provocation were not included to exclude a possible influence of provocation on earn trials.

#### 2.7.2 fMRI: group analyses

Each task run was taken into the group analyses along with the transformations to standard MNI152 space to generate group-level contrasts. To constrict the analyses to our research question on the cerebellum as region-of-interest, we used a binary mask of the cerebellar grey matter from the spatially unbiased atlas template (SUIT; Diedrichsen et al., 2009) normalised to MNI152 space with FLIRT. Secondary whole brain analyses without masking are reported in the Supplementary Material, to put our findings in the context of previous research.

For group analyses, mixed-effects modelling was performed with FLAME 1 in FEAT (Smith et al., 2004; Woolrich et al., 2004). At the group level, one-sample *t*-tests were applied for our contrasts Provocation > Earn and Steal > Earn. Group level contrast images for Provocation > Earn were cluster thresholded at *Z* = 3.1 (*p* < 0.001) and corrected for Family-Wise Error (FWE) at *p* = 0.05 (Worsley, 2003). Due to behavioural flexibility during the PSAP, not all participants stole from their opponents. For the group analyses of Steal > Earn, we only included participants that stole at least 10 times during the task to provide sufficient data (*n* = 19). Due to the smaller sample size, a more liberal cluster-based threshold was applied (*p *< 0.005 instead of *p* < 0.001 to balance Type I and II errors; Lieberman & Cunningham, 2009). Results were visualised in standard sagittal, coronal, and axial slices, as well as flatmaps rendered in SPM12’s SUIT toolbox (Diedrichsen et al., 2009).

#### 2.7.3 Associations between cerebellar activation and measures of aggression

In the whole sample (Provocation > Earn), associations between cerebellar activation, personality characteristics, and the T/C ratio were explored. For each cluster, the maximal *Z* value from each participant was extracted from the group-level analysis. These values were correlated with the total BPA and BIS-11 scores, change in SA scores, T/C ratio and pre-scan testosterone and cortisol levels using Pearson correlations for parametric data, and Spearman correlations for non-parametric data. An FDR-corrected *p < *0.05 was considered significant (Benjamini & Hochberg, 1995), with additional corrections for multiple cerebellar clusters if necessary.

#### 2.7.4 Data reduction and additional behavioural analyses

Behavioural analyses were performed with R version 3.6.0 in RStudio version 1.2.1335 for Windows (RStudio Team, 2018). Testosterone levels were *Z*-transformed per sex to account for sex differences in testosterone. Additionally, the ratio between testosterone and cortisol levels (T/C) was calculated by dividing *Z*-transformed testosterone levels by cortisol levels. For the PSAP, aggressive behaviour was calculated as the number of steal trials divided by the number of total button presses.

To assess whether there was an increase in SA scores after the PSAP in the MRI scanner compared to pre-task levels, a paired Wilcoxon signed rank test was conducted, where *p* < 0.05 (one-sided) was considered a significant increase. We checked whether participants who did not steal often (<10 times) and participants who stole at least 10 times in two runs scored differently on trait aggression or impulsivity (BPA/BIS-11 scores) with a two-sample *t*-test. Within the group that used aggressive responses during the PSAP (i.e., participants who stole at least 10 times), aggressive behaviour was correlated with trait aggression and impulsivity (BPA and BIS-11 scores) and percentage changes in state anger using Pearson correlations for parametric data and Spearman correlations for non-parametric data. Exploratively, subscales of the BPA and BIS-11 questionnaires were correlated with aggressive behaviour if there was a significant association with total scores, to take the multidimensionality of these constructs into account (Patton et al., 1995). For all behavioural analyses, individual observations were considered outliers if they deviated by at least three standard deviations from the group mean. For these tests, a *p*-value < 0.05 (two-sided) was considered significant. For further behavioural analyses on hormone levels, see Supplementary Section 1.

## 3 Results

### 3.1 Study population

Demographic characteristics of the study population are summarised in Table 1. Two participants had undetectable (<0.5 nmol/L) levels of cortisol and their data were not taken into analyses of hormone levels. Furthermore, one participant did not fill in the state anger questionnaire prior to the scanning session and was also excluded. In the remaining group (*n* = 28), state anger scores were significantly higher after the scan compared to before (*Z* = -1.93, *p* = 0.027).

**Table 1.** Characteristics of the study population.

	Healthy volunteers (*n*=29) Mean	Range
Demographics		
Age (years)	23.0 ± 3.2	18–32
Male (n)	15 (51.7%)	
Hormone levels		
Testosterone (pmol/L)		
Males	230 [218–272]	201–338
Females	107 [61–130]	46–198
Cortisol (nmol/L)		
Males	2.4 [1.5–3.5] ^†^	1.2–5.2
Females	3.1 [2.0–6.3]	0.8–21.4
T/C ratio		
Males	0.11 [0.08–0.14] ^†^	0.04–0.21
Females	0.03 [0.02–0.05]	0.00–0.09
Questionnaires		
SA pre-scan	10.0 [10.0–11.0] ^‡^	10–15
SA post-scan	11.0 [10.0–11.0]	10–16
BPA physical aggression	16.0 [13.0–20.0]	10–29
BPA verbal aggression	10.0 [9.0–14.0]	6–19
BPA anger	11.0 [10.0–16.0]	8–24
BPA hostility	16.0 [13.0–19.0]	8–29
BPA total	59.0 [48.0–63.0]	40–83
BIS-11 attentional impulsivity	18.0 [15.0–19.0]	9–23
BIS-11 motor impulsivity	21.0 [19.0–24.0]	14–26
BIS-11 non-planning impulsivity	23.0 [19.0–26.0]	16–34
BIS-11 total	62.0 [53.0–68.0]	44–78

Data are presented as mean ± standard deviation or median [interquartile range (IQR)] for continuous variables and as number (percentage of total) for categorical variables. Displayed steroid hormone levels are not standardised. ^†^ data available in *n* = 27; ^‡^ data available in *n* = 28. Abbreviations: BIS-11 = Barratt Impulsiveness Scale; BPA = Buss Perry Aggression questionnaire; SA = State Anger, T/C = Testosterone/Cortisol.

### 3.2 PSAP behaviour

PSAP behaviour is summarised in Table 2. During the task, provocations occurred on average in every five trials (19.6%) and a total of 10 times per run. On average, participants stole 5.8 times [IQR 2.0— 7.8] relative to total button presses. From the 29 participants, 10 participants stole less than 10 times.

**Table 2.** Behaviour during the Point Subtraction Aggression Paradigm.

	Average number of times (per 9 minute run)
Earn	31.5 [27.0–36.0]
Steal	8.5 [2.5–11.0]
Steal without preceding provocation (% of steal)	80.0 [75.0–88.5] ^‡^
Steal after provocation (% of steal)	20.0 [11.5–25.0] ^‡^
Protect	15.0 [11.0–18.0]
Provocations	10.0 [8.0–11.5]
Trials	55.5 [50.5–59.5]

Data are presented as median [IQR]. ^‡^ data on types of steals available in *n* = 25, which includes everybody that stole at least once.

### 3.3 Cerebellar activation

**Fig. 2.**
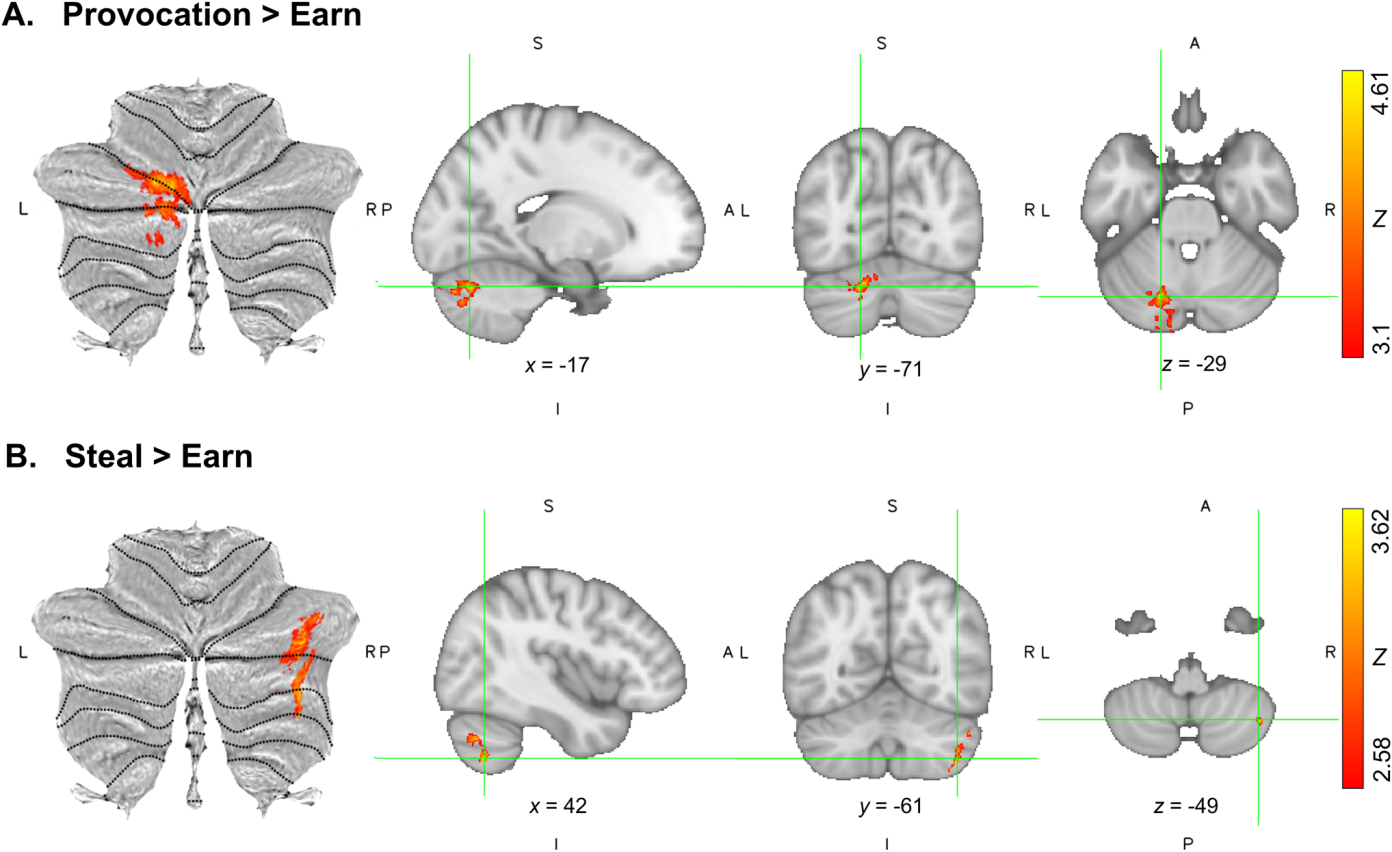
Cerebellar activation during the Point Subtraction Aggression Paradigm. (A) Left cerebellum in the Provocation > Earn contrast (*n* = 29). Peak activation was located at MNI coordinates *x* = -17, *y* = -71, *z* = -29. Cluster-threshold *Z* = 3.1. (B) Right cerebellum in the Steal > Earn contrast (*n* = 19). Peak activation was located at MNI coordinates *x* = 42, *y* = -61, *z* = -49. Cluster-threshold *Z=* 2.576. Visualisations focused at *Z* max on a SUIT flatmap and sagittal, coronal, and axial slices. All images are displayed in neurological orientation.

When points got stolen from the participants (Provocation > Earn), participants showed activation in the left posterior cerebellar lobe (cluster peak in left lobule VI/Crus I) (Table 3, Fig. 2). Data from the 19 participants who stole at least 10 times were taken into further analyses (i.e., Steal > Earn). For Steal > Earn, activation was present in the right posterior lobe (cluster peak in right Crus II/lobule VIIb) (Table 3, Fig. 2). Whole brain findings are reported in the Supplementary Material.

**Table 3.** Task activation in the Point Subtraction Aggression Paradigm in the cerebellum.

Anatomical region peak ^a^	*p*-value (FWE-corrected)	Cluster size (voxels)	*Z* max	Peak MNI coordinates		
				*x*	*y*	*z*
Provocation > Earn						
Left Crus I / Left VI	<0.001	1766	4.61	-17	-71	-29
Steal > Earn *						
Right Crus II / Right VIIb	<0.001	634	3.62	42	-61	-49

a Cerebellar Atlas in MNI152 space after normalisation with FLIRT (Diedrichsen et al., 2009).

* 19 of the 29 participants included in this contrast.

Abbreviations: FWE = Family Wise Error; MNI = Montreal Neurological Institute.

### 3.4 Cerebellar activation-behaviour analyses

Neither the max *Z* scores of the Provocation > Earn contrast (*n* = 29) nor the max *Z* scores from the Steal > Earn contrast (*n* = 19) were significantly associated with any of the behavioural or hormonal measures (*p*s = 0.978, see Table S1).

### 3.5 Additional behavioural analyses

**Fig. 3.**
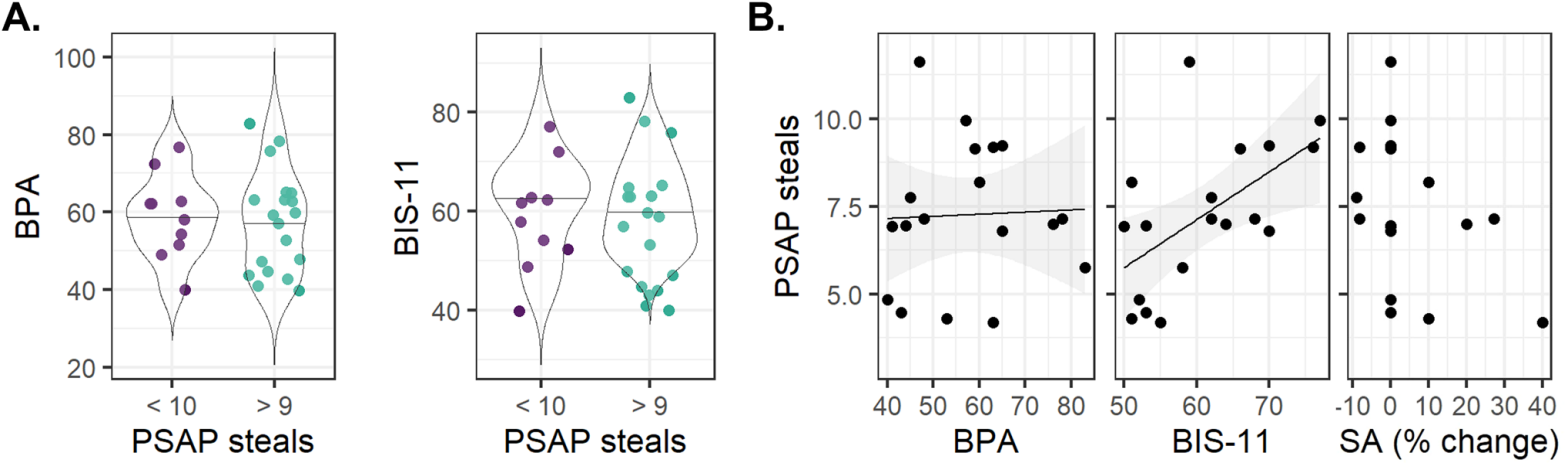
The association between PSAP stealing behaviour and questionnaire scores. (A) No difference in BPA and BIS-11 scores between participants that stole less than 10 times and at least 10 times. (B) PSAP steals (relative to total button presses) correlations with BPA and BIS-11 total scores and change in SA scores within the group that stole at least 10 times. Abbreviations: BIS-11 = Barratt Impulsiveness Scale; BPA = Buss-Perry Aggression questionnaire, PSAP = Point Subtraction Aggression Paradigm, SA = State Anger.

Prior to the behavioural analyses, one outlier was removed for more PSAP relative steals and one outlier was removed for a high change in SA scores. There was no association between BPA/BIS-11 scores and the tactic employed during the task. Participants that employed a tactic of primarily earning and protecting (i.e., total number of steals < 10) did not show a difference in BPA (*t*_27_ = -0.30, *p* = 0.765) or BIS-11 (*t*_27_ = -0.20*, p* = 0.843) scores compared to the group that stole at least 10 times (Fig. 3A). Within the group of people that used stealing (>9 times) in their tactic (*n* = 19), there was no correlation between the number of steals and BPA scores (*r=* 0.04, *p_FDR_* = 0.879; Fig. 3B). Higher BIS-11 scores, however, were correlated with more steals (*r=* 0.62, *p_FDR_* = 0.018; Fig. 3B). Exploratory post-hoc analyses showed that this relation appeared to be driven by the non-planning impulsivity subscale (*r=* 0.69, *p* = 0.002; motor impulsivity: *p* = 0.176; attentional impulsivity: *p=* 0.312). Finally, the percentage change in SA scores was marginally correlated with the number of steals during the PSAP, but this was not significant after FDR correction (*ρ* = -0.38, *p_FDR_* = 0.205) (Fig. 3B).

## 4 Discussion

The aim of the present study was to investigate cerebellar activation in response to provocation and during aggressive behaviour. Results showed left posterior cerebellar activation when provocations occurred, while right posterior cerebellar activation was observed when participants engaged in aggressive behaviour.

The left hemispheric cluster of cerebellar activation during provocation with a paravermal peak in lobule VI/Crus I concurs with previous meta-analyses that showed activation of left Crus I and II during passive processing of negative emotions, including anger, disgust, and sadness (E et al., 2014; Klaus & Schutter, 2021; Pierce et al., 2022). Furthermore, another study in healthy volunteers confirmed the involvement of the left posterior cerebellar areas, mainly Crus I and II, during emotion processing (King et al., 2019). In emotion processing, arousal and negative valence are suggested to involve left lobule VI (i.e., valence), left Crus II and vermal lobules VI and VIIIa (i.e., arousal), and left lobules V and Crus I (i.e., arousal-valence interaction) (Styliadis et al., 2015). These areas align with our findings in left lobule VI/Crus I-II. It is conceivable that the left posterior cerebellum plays a role in regulating arousal through connections with the reticular system via the cerebellar fastigial nuclei. These connections with the fastigial nuclei have been shown to originate from both the vermis and hemispheric lobules VI and Crus I-II in mice (Fujita et al., 2020). In processing valence, the cerebellum can communicate with the amygdala (Schienle & Scharmüller, 2013), hypothalamus (Moulton et al., 2011), and mPFC (Etkin et al., 2011; Krienen & Buckner, 2009) through mono- and/or polysynaptic connections. Furthermore, different emotions may be associated with distinct cerebellar patterns. For example, disgust was associated with activation of a left lobule VI cluster slightly anterior to our current cluster (Baumann & Mattingley, 2012). Provocation can also elicit an aversive state (e.g., moral disgust), an emotional response to offensive stimuli, which has previously been associated with decreased aggressive responses (Bondü & Richter, 2016). In addition to arousal and affective responses, provocations are also considered to be threatening. The left posterior Crus I-II could contribute to the threat detection circuit via the fastigial nuclei and PAG, through connections that have been evidenced in mice (Fujita et al., 2020), and thus initiate fight, flight, or freeze responses (Roelofs, 2017). Provocation may also increase the tendency to physically avoid confrontation by attending away from threatening stimuli such as angry faces, stepping away, or pushing a lever away (Roelofs et al., 2008; Stins et al., 2011; van Honk et al., 1998). The posterior cerebellum together with the prefrontal cortex (PFC) has been proposed to be a part of a cerebello-cortical system involved in approach- and avoidance-related motivation and emotion (Schutter, 2020). According to this idea, avoidance-related behaviour is lateralised to the left posterior cerebellum and right PFC (Kelley et al., 2017; Schutter, 2020). In line with this argumentation, left Crus I and II were shown to be structurally connected to the right prefrontal cortex in primates (Kelly & Strick, 2003; Middleton & Strick, 2001). Furthermore, avoidant behaviour is also a symptom of fear and anxiety disorders (Bögels et al., 2010), which also show altered activation in the cerebellum (Ernst et al., 2019; Hoppenbrouwers et al., 2008; Moreno-Rius, 2018). In sum, in line with previous findings on negative emotion processing and avoidance-related behaviour (e.g., Klaus & Schutter, 2021; Schutter, 2020), left posterior cerebellar activation was observed when participants were provoked.

Right cerebellar task activation during stealing from the opponent is in line with previous meta-analytic evidence reporting activation of the right rostral-posterior regions, including Crus I-II and lobule VIIb, during aggressive behaviour (Klaus & Schutter, 2021). In further support, a recent volumetry study in healthy volunteers showed that grey matter volumes of the right posterolateral lobules VIIb and VIIIa were correlated with aggressive and impulsive personality traits (Wolfs, Klaus, et al., 2023). Complementary to our findings in the left posterior cerebellar lobe, we speculate that the right cerebellar hemisphere may be involved in the approach system through its contralateral connections with the left PFC (Middleton & Strick, 2001). Previous research has shown that relative left-to-right dominant PFC activity is associated with a person’s increased tendency for approach-related behaviour, which is associated with anger and aggressive behaviour (Harmon-Jones & Sigelman, 2001; Kelley et al., 2017; Schutter & Harmon-Jones, 2013). The right posterior cerebellum may thereby be involved in increasing or decreasing the likelihood to approach and behave aggressively (Schutter, 2020). In addition to the lateral PFC, Crus I-II are connected to the default mode network hubs, including the mPFC (Buckner et al., 2011; Krienen & Buckner, 2009). The mPFC, in turn, has also been found to be activated in laboratory aggression paradigms (Chen et al., 2021; da Cunha-Bang et al., 2017; Kaltsouni et al., 2021; Skibsted et al., 2017) and is suggested to be involved in the subjective experience of conflict between retaliation and non-aggressive responses (Repple et al., 2017). In aggressive veterans, the medial orbitofrontal cortex also showed altered functional connectivity with the DCN, the collection of areas that relays information from the cerebellar cortex to other parts of the brain (Wolfs, van Lutterveld, et al., 2023). Furthermore, functional connections exist between the right posterior cerebellum and the fronto-parietal network involved in response inhibition and executive functioning (Buckner et al., 2011; Habas et al., 2009; Osada et al., 2019). Interestingly, participants with higher non-planning impulsivity scores (e.g., not planning ahead and saying things without thinking) showed increased aggressive behaviour during the PSAP in the current study. This adds to the idea that the right posterolateral lobules may be involved in impulsive behaviour (Wolfs, Klaus, et al., 2023). In addition to approach-related motivation and impulsivity, aggressive behaviour is arguably associated with a higher reward-drive and lower punishment sensitivity (Megías-Robles et al., 2022). Our right posterior findings, however, do not overlap with a recent meta-analysis on reward-processing in the cerebellum (Kruithof et al., 2023) which found involvement of vermal VI/Crus I during reward outcome and the posterior vermis, bilateral lobules I-VI, and lateral left Crus I during reward anticipation. In the present paradigm, it was difficult to pinpoint the rewarding aspect of aggressive behaviour, as earning points in itself already serves as a reward. Further studies with aggression paradigms that do not use reward as the main task goal for participants may be considered in future research. In sum, in line with the lateralisation of the approach-avoidance system, right posterior cerebellar activation was observed when participants behaved aggressively. The specific underlying functional system in play, however, remains to be elucidated.

Contrary to our expectations, no evidence was found for activation of the vermis during provocation or aggressive behaviour. The vermis is thought to be part of the “limbic cerebellum” and has been found to play a role in processes linked to the experience and regulation of emotions (Adamaszek et al., 2017; E et al., 2014; Frazier et al., 2022; Guell et al., 2018; Leggio & Olivito, 2018). It is involved in arousal and autonomic activation through its connections to subcortical brain structures, such as the amygdala, reticular formation, and hypothalamus, which regulate the sympathetic and parasympathetic branches of the central nervous system arousal and autonomic responses (Schmahmann, 2000; Styliadis et al., 2015). For example, activity attributed to arousal has been found in vermal lobules VI and VIIIa (Styliadis et al., 2015). Owing to the nature of provocation in the PSAP, somatic responses to stealing and preparing the body to fight or flight may have been limited. Provocations in this study are relatively mild and might elicit mainly a cognitive response, since a defensive response (fight-or-flight) was neither warranted nor physically possible. In future studies, vermal activation may be elicited through the use of stronger provocations such as loud noises, electric shocks, or proximal (e.g., inescapable) threats (e.g., Faul et al., 2020). Additionally, administering a frustration task before the experimental task (e.g., Hortensius et al., 2011) may increase baseline state anger levels and thus provide more insights into the effect of heightened emotional responses on aggressive behaviour. Finally, adding physiological measures of arousal, such as heart rate and pupil dilation, may be interesting to examine if cerebellar activation can be more directly linked to sympathetic arousal in the context of provocation and aggressive behaviour.

In accordance with the goal of the PSAP to elicit aggressive behaviour and frustration (Cherek et al., 1997; Geniole et al., 2017), self-reported state anger was higher after the task as compared to baseline. Because aggressive behaviour during the PSAP was a voluntary act, different tactics were employed throughout the task regardless of trait aggression or impulsivity scores. Aggressive behaviour costs effort and interferes with the participants’ (primary) goal of collecting points and earning money. Participants (*n* = 10) who did not frequently steal (<10 times) may have had a stronger motivation to “win”: They reported that earning and protecting was the most efficient way to earn as many points as possible (i.e., the group that did not frequently steal scored on average 23.2 ± 5.7 points, whereas the other group scored 19.5 ± 4.2 points (post-hoc difference: *t_27_* = 1.80, *p* = 0.094)). Exploratively, between these groups there was no difference in cerebellar activation when participants were provoked, suggesting that the initial reaction to provocations was similar regardless of subsequent behavioural responses (see Supplementary Section 5). Statistical power for this post-hoc analysis, however, was low and further studies are needed to investigate this difference. In addition, the PSAP could be adapted to facilitate immediate stealing whilst being provoked without finishing the current trial. This can provide additional information on aggressive intentions and offer a more naturalistic scenario to respond to provocations.

No evidence was found that the T/C ratio was associated with aggressive behaviour during the PSAP. Furthermore, no correlations between cerebellar activation, behaviour, and steroid hormones were observed. As mentioned earlier, the mild provovations during the task may not have been sufficient to evoke physiological responses to provocation. Our findings also add to the idea that the link between testosterone, cortisol, and aggression is highly complex and that (social) context plays an important role (for reviews, see Geniole et al., 2017, 2020). The T/C ratio may be more difficult to capture in tasks that include provocations, because the association between T/C ratio and aggressive behaviour was shown to be weaker or absent when provoked (Geniole et al., 2011; Manigault et al., 2019), although opposite relations have also been reported (Denson et al., 2013). Whilst looking at the hormones separately, lower testosterone levels were associated with higher BIS-11 non-planning impulsivity scores (Supplemental Section 1). Speculatively, our findings on the association between higher testosterone levels and lower impulsivity scores could be linked to the involvement of testosterone in goal-directed behaviour, risk aversive strategies, and maintaining social status (Heany et al., 2018; van Honk et al., 2016). In line with this, prior studies provide inconclusive evidence on the association between aggressive behaviour in the PSAP and testosterone levels, but also emphasise the importance of looking at moderating factors (e.g., sex, anxiety levels, sleep deprivation) in more detail (Geniole et al., 2017). To better establish the role of testosterone and cortisol within the cerebellar framework of anger and aggressive behaviour, larger sample sizes and more sensitive methods to assess steroid hormone levels are necessary. Finally, administration studies (e.g., Goetz et al., 2014; Hermans et al., 2008) may be another way to examine the proposed links between steroid hormones, the cerebellum, provocation, and aggressive behaviour.

In conclusion, provocation and aggressive behaviour were linked to two spatially distinct regions of activation in the human cerebellum. Our findings provide evidence for the involvement of distinct non-motor related cerebellar areas during both provocation and aggressive behaviour and add to the growing recognition of the posterior cerebellar regions in emotion- and cognition-dedicated processes.

## Data and Code Availability

Data and code are stored in Yoda, a data management system hosted by Utrecht University, and can be accessed upon request at https://doi.org/10.24416/UU01-E8Z2LQ.

## Author Contributions

Elze M.L. Wolfs: Conceptualisation; Formal analysis; Investigation; Methodology; and Writing—original draft and preparation; Wietske van der Zwaag: Methodology; Investigation; and Writing—review and editing; Nikos Priovoulos: Methodology; Investigation; and Writing—review and editing; Jana Klaus: Conceptualisation; Methodology; Supervision; and Writing—review and editing; and Dennis J.L.G. Schutter: Conceptualisation; Funding acquisition; Methodology; Supervision; and Writing—review and editing.

## Funding

This work was supported by the Dutch Research Foundation (NWO, VI.C.181.005).

## Declaration of Competing Interest

None.

## Supplementary Materials

Supplementary material for this article is available with the online version here: https://doi.org/10.1162/imag_a_00044.

## Supplementary Material

Supplementary Material
