# Supplementary material for: The cerebellum during provocation and aggressive behaviour: A 7 T fMRI study
Source: Imaging Neurosci (Camb). 2023 Dec 14;1:imag-1-00044. doi: 10.1162/imag_a_00044 (PMC12007546; doi:10.1162/imag_a_00044)
Supplement: Supplementary Material [file imag_a_00044-supp.pdf]

Supplement

1. Additional behavioural analyses

Spearman correlations were used to examine the associations between steroid hormone levels and behaviour during the Point Subtraction Aggression Paradigm (PSAP), scores on the Buss-Perry Aggression questionnaire (BPA)/Barratt Impulsiveness Scale (BIS-11), and change in state anger (SA) scores. FDR-corrected *p*-values < 0.05 are considered significant (Benjamini & Hochberg, 1995). Results of the correlational analyses are presented in Figure S1A. Within the group of participants that had detectable cortisol levels (> 0.5 nmol/L, $n = 27$), one participant was excluded each for cortisol and the testosterone/cortisol ratio as outlier (> $M + 3*SD$, $n = 26$). For change in state anger scores, one participant was excluded as outlier.

BIS-11 total scores were negatively associated with pre-scan testosterone levels ($\rho = -0.57$, $p_{FDR} = 0.015$, $n = 29$) (Figure S1A-B). Exploratory analysis showed that from the BIS-11 subscales non-planning and attentional impulsivity were negatively associated with testosterone levels ($\rho = -0.56$, $p = 0.002$; $\rho = -0.46$, $p = 0.012$).

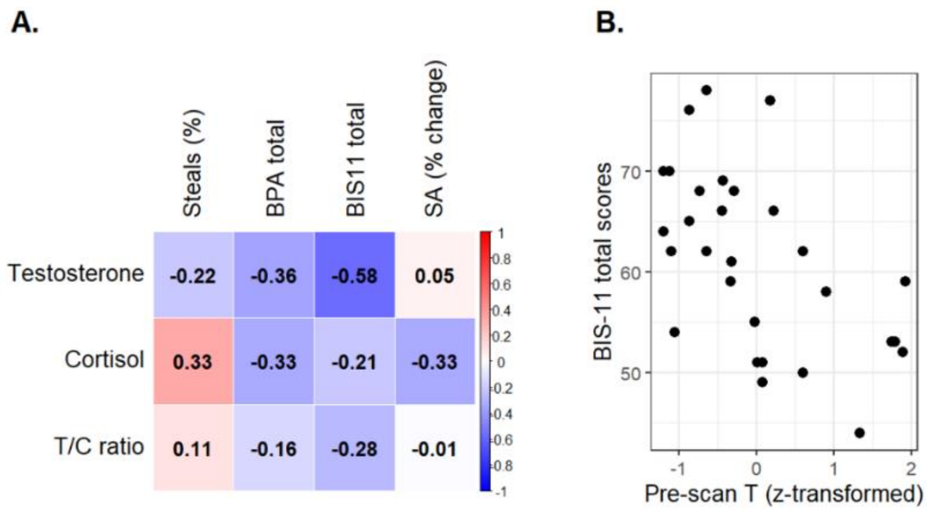

Figure S1. Spearman correlations between steroid hormone levels and measures of aggressive behaviour, impulsivity and anger. [A] Correlation rho, as represented by the colours and numbers, between hormone levels and measures of aggression/impulsivity. Steals represent the number of steals during the PSAP relative to total button presses. [B] The significant negative non-parametric correlation between the pre-scan testosterone levels and BIS-11 impulsivity scores. Abbreviations: BPA = Buss-Perry Aggression Questionnaire; BIS-11 = Barratt Impulsiveness Scale; C = Cortisol; PSAP = Point Subtraction Aggression Paradigm; SA = State Anger; T = Testosterone.

2. Cerebellar activation-behaviour analyses

Table S1. Brain-behaviour correlations between cerebellar activation levels, trait aggression/impulsivity scores and steroid hormones.

	BPA total	BIS-11 total	SA (% change)	Pre-scan T	Pre-scan C	Pre-scan T/C ratio
Provocation > Earn	r = -0.168	r = -0.233	ρ = 0.135	ρ = -0.005	ρ = -0.014	ρ = 0.077
Max Z score	p = 0.978	p = 0.978	p = 0. 978‡	p = 0.978	p = 0. 978†	p = 0.978†
Steal > Earn *	r = -0.129	r = -0.053	ρ = 0.113	ρ = -0.219	ρ = -0.054	ρ = -0.034
Max Z score	p = 0.978	p = 0.978	p = 0.978°	p = 0.978	p = 0.978°	p = 0.978°

* 19 of the 29 participants. ‡ data available in n = 28; † data available in n = 26. ° data available in n = 18.
All p-values are FDR corrected. Abbreviations: BIS-11 = Barratt Impulsiveness Scale; BPA = Buss Perry Aggression questionnaire; C = Cortisol; SA = State Anger; T = Testosterone.

3. Provocation > Earn: whole brain analysis

For Provocation > Earn and Earn > Provocation whole brain results are shown in Table S2 and Figure S2. Provocation compared to earning a point was linked to higher activation in the left lateral occipital cortex and precuneus, in line with findings reported in previous studies using comparable aggression-related paradigms. Activation in these brain regions are associated with processes involved in attention and social cognition/self-consciousness (Fanning et al., 2017; Wong et al., 2019). Furthermore, the right inferior/middle frontal gyrus is implicated in response inhibition (Aron et al., 2004). Compared to provocation, earning a point led to increased activation in the planum temporale, anterior cingulate cortex, insula and putamen. These regions contribute to reward processing and the salience network (Seeley et al., 2007).

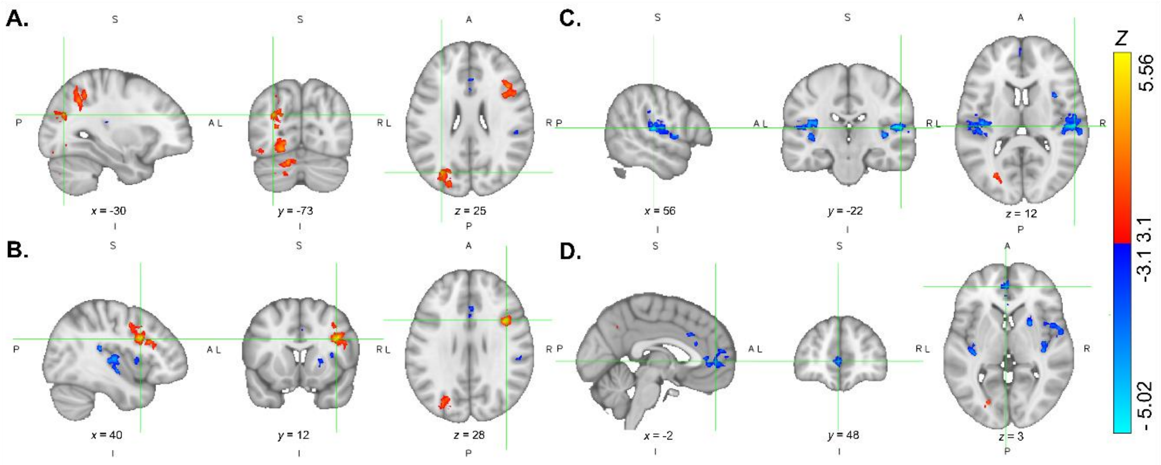

Figure S2. Provocation vs Earn contrast, four slices visualised from the whole brain analyses. [A] Provocation > Earn peak at x = -30, y = -73, z = 25. [B] Provocation > Earn peak at x = 40, y = 12 , z = 28. [C] Earn > Provocation peak (blue) at x = 56, y = -22, z = 12. [D] Earn > Provocation peak (blue) at x = -2, y = 48, z = 3.

Table S2. Task-related whole brain grey matter clusters for Provocation > Earn

Anatomical Region peak location [a]	*P*-value (FWE-corrected)	Cluster size (voxels)	*Z* max	Peak MNI coordinates *x*	*y*	*z*
Provocation > Earn						
Left Superior Lateral Occipital Cortex	< 0.001	4497	4.77	-30	-73	25
Right Inferior/Middle Frontal Gyrus	< 0.001	2405	5.56	40	12	28
Left Superior Parietal Lobule	< 0.001	1986	4.46	-30	-57	41
Left Crus I	< 0.001	1766	4.61	-17	-71	-29
Right Precuneus	0.004	448	4.02	8	-64	46
Left Inferior Lateral Occipital Cortex	0.030	317	4.16	-36	-85	-18
Earn > Provocation						
Right Planum Temporale	< 0.001	4882	4.93	56	-22	12
Left Central Opercular Cortex / Heschl's Gyrus / Insular cortex	< 0.001	2889	5.02	-41	-20	13
Paracingulate Gyrus	< 0.001	1287	4.17	-2	48	3
Cingulate Gyrus, anterior division	< 0.001	564	4.72	0	21	33
Right Putamen	0.004	445	4.34	24	8	3

[a] Atlas: (sub)cortical areas: Harvard-Oxford (Sub)Cortical Structural Atlas (Desikan et al., 2006); cerebellar areas: Cerebellar Atlas in MNI152 space after normalisation with FLIRT (Diedrichsen et al., 2009).

In the whole brain analysis, a $Z > 3.1$ threshold did not show activation of the DCN, the main output structure of the cerebellum. High iron content of the DCN causes a weaker signal compared to the surrounding white and grey matter (Diedrichsen et al., 2011). At a lower threshold ($Z > 2.3$) deactivation of the right dentate nucleus was observed for Provocation > Earn, see Figure S3.

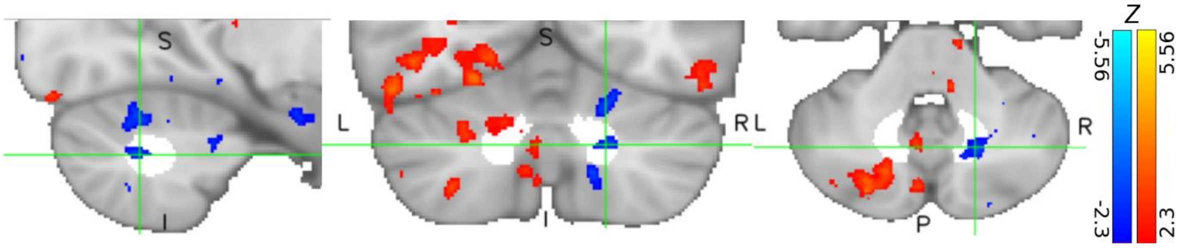

Figure S3. Deactivation in the right dentate nucleus (in white) for the Provocation > Earn contrast thresholded at *Z* > 2.3

4. Steal > Earn: whole brain analysis

For Steal > Earn and Earn > Steal whole brain results are shown in Table S3 and Figure S4. Stealing a point showed stronger activation in the left lateral occipital cortex, left occipital fusiform gyrus and precuneus. Activation of the occipital regions have previously also been found during provocation subserving attentional and visual information processing (Fanning et al., 2017). In studies on aggression the precuneus has been associated with social cognition/self-consciousness (Fanning et al., 2017; Wong et al., 2019).

Table S3. Task-related whole brain grey matter clusters for Steal > Earn

Anatomical Region peak location [a]	*P*-value (FWE-corrected)	Cluster size (voxels)	*Z* max	Peak MNI coordinates *x*	*y*	*z*
*Steal > Earn **						
Left Superior Parietal Lobule / Superior Lateral Occipital Cortex	< 0.001	2842	4.22	-31	-58	54
Left Occipital Fusiform Gyrus	< 0.001	1415	4.2	-23	-76	-5
Right Precuneus	< 0.001	756	4.38	2	-60	45
Right Crus II	0.002	634	3.62	42	-61	-49
Left Lingual Gyrus	0.003	626	4.1	-3	-87	-12
*Earn > Steal **						
Right Precuneus	< 0.001	2302	3.95	13	-57	9
Left Planum Temporale	< 0.001	1311	4.24	-48	-28	11
Anterior Cingulate Gyrus	< 0.001	1193	3.86	-1	8	34
Right Central Opercular Cortex	< 0.001	986	4.38	60	-9	8
Right Parietal Operculum Cortex	< 0.001	814	4.3	42	-32	18
Right Occipital Pole	< 0.001	752	3.96	25	-98	11
Right Inferior Frontal Gyrus	0.023	461	3.67	36	32	4
Left Insular Cortex	0.026	454	3.74	-37	-23	3
Left Superior Temporal Gyrus	0.047	411	3.72	-54	-16	-7

[a] Atlas: (sub)cortical areas: Harvard-Oxford (Sub)Cortical Structural Atlas (Desikan et al., 2006); cerebellar areas: Cerebellar Atlas in MNI152 space after normalisation with FLIRT (Diedrichsen et al., 2009). * 19/29 participants.

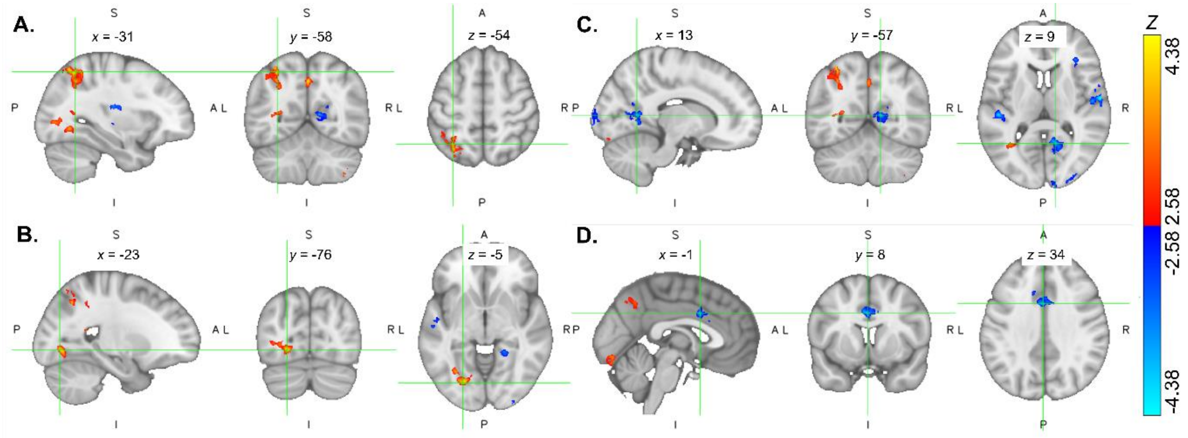

Figure S4. Steal vs Earn contrast, four slices visualised from the whole brain analyses. [A] Steal > Earn peak at *x* = -31, *y* = -58, *z* = -54. [B] Steal > Earn peak at *x* = -23, *y* = -76, *z* = -5. [C] Earn > Steal peak (blue) at *x* = 13, *y* = -57, *z* = 9. [D] Earn > Steal peak (blue) at *x* = -1, *y* = 8, *z* = 34.

5. Provocation > Earn group difference

An exploratory post-hoc analysis was performed to see if there was a difference in cerebellar activation for Provocation > Earn, for participants that stole > 9 times and participants that stole < 10 times by use of a two sample *t*-test. Again, mixed effects modelling was performed with FLAME 1 in FEAT (Smith et al., 2004; Woolrich et al., 2004) and group level contrast images were cluster thresholded at $Z = 3.1$ ($p < 0.001$) and corrected for Family-Wise Error (FWE) at $p = 0.05$ (Worsley, 2001). Between the two groups, we did not find any significant cluster in the cerebellum ($Z > 3.1$).

Supplemental references

Aron, A. R., Robbins, T. W., & Poldrack, R. A. (2004). Inhibition and the right inferior frontal cortex. *Trends in Cognitive Sciences*, 8(4), 170-177. doi:10.1016/j.tics.2004.02.010

Benjamini, Y., & Hochberg, Y. (1995). Controlling the False Discovery Rate: A Practical and Powerful Approach to Multiple Testing. *Journal of the Royal Statistical Society: Series B (Methodological), 57*(1), 289-300. doi:10.1111/j.2517-6161.1995.tb02031.x

Desikan, R. S., Ségonne, F., Fischl, B., Quinn, B. T., Dickerson, B. C., Blacker, D., Buckner, R. L., Dale, A. M., Maguire, R. P., & Hyman, B. T. (2006). An automated labeling system for subdividing the human cerebral cortex on MRI scans into gyral based regions of interest. *NeuroImage, 31*(3), 968-980. doi:10.1016/j.neuroimage.2006.01.021

Diedrichsen, J., Balsters, J. H., Flavell, J., Cussans, E., & Ramnani, N. (2009). A probabilistic MR atlas of the human cerebellum. *NeuroImage, 46*(1), 39-46. doi:10.1016/j.neuroimage.2009.01.045

Diedrichsen, J., Maderwald, S., Küper, M., Thürling, M., Rabe, K., Gizewski, E. R., . . . Timmann, D. (2011). Imaging the deep cerebellar nuclei: A probabilistic atlas and normalization procedure. *NeuroImage*, 54(3), 1786-1794. doi:10.1016/j.neuroimage.2010.10.035

Fanning, J. R., Keedy, S., Berman, M. E., Lee, R., & Coccaro, E. F. (2017). Neural Correlates of Aggressive Behavior in Real Time: a Review of fMRI Studies of Laboratory Reactive Aggression. *Current Behavioral Neuroscience Reports, 4*(2), 138-150. doi:10.1007/s40473-017-0115-8

Seeley, W. W., Menon, V., Schatzberg, A. F., Keller, J., Glover, G. H., Kenna, H., . . . Greicius, M. D. (2007). Dissociable Intrinsic Connectivity Networks for Salience Processing and Executive Control. *The Journal of Neuroscience, 27*(9), 2349-2356. doi:10.1523/jneurosci.5587-06.2007

Smith, S. M., Jenkinson, M., Woolrich, M. W., Beckmann, C. F., Behrens, T. E. J., Johansen-Berg, H., Bannister, P. R., De Luca, M., Drobnjak, I., Flitney, D. E., Niazy, R. K., Saunders, J., Vickers, J., Zhang, Y., De Stefano, N., Brady, J. M., & Matthews, P. M. (2004). Advances in functional and structural MR image analysis and implementation as FSL. *NeuroImage, 23*, S208-S219. doi:10.1016/j.neuroimage.2004.07.051

Wong, T. Y., Sid, A., Wensing, T., Eickhoff, S. B., Habel, U., Gur, R. C., & Nickl-Jockschat, T. (2019). Neural networks of aggression: ALE meta-analyses on trait and elicited aggression. *Brain Structure and Function, 224*(1), 133-148. doi:10.1007/s00429-018-1765-3

Woolrich, M. W., Behrens, T. E. J., Beckmann, C. F., Jenkinson, M., & Smith, S. M. (2004). Multilevel linear modelling for FMRI group analysis using Bayesian inference. *NeuroImage, 21*(4), 1732-1747. doi:10.1016/j.neuroimage.2003.12.023

Worsley, K. J. (2001). Statistical analysis of activation images. *Functional MRI: An introduction to methods,* 14(1), 251-270.
